# The Long Fox Memorial Lecture: The Partnership between Anæsthesia and Surgery

**Published:** 1931

**Authors:** Arthur L. Flemming

**Affiliations:** Consulting Anæsthetist to Bristol Royal Infirmary


// The Bristol
Medico-Chirurgical Journal

*"Scire est nescire, nisi id me
Scire alius sciret."*

WINTER, 1931.

### THE LONG FOX MEMORIAL LECTURE:

DELIVERED IN THE UNIVERSITY OF BRISTOL ON OCTOBER 27TH, 1931.

THE VICE-CHANCELLOR (Dr. T. LOVEDAY, M.A., LL.D.) *in the Chair.*

BY

ARTHUR L. FLEMMING, M.B., CH.B., L.R.C.P., M.R.C.S.,
*Consulting Anæsthetist to Bristol Royal Infirmary.*

ON

### THE PARTNERSHIP BETWEEN ANÆSTHESIA AND SURGERY.

---

As a product of the Bristol Medical School, naturally I am fully sensitive of the privilege which the Committee have bestowed upon me, and I wish to offer to them my sincere thanks for inviting me to deliver this address. Nevertheless, I am painfully alive to my responsibility in undertaking so active a part in this the twentieth annual meeting held to honour

P

the name of the late Dr. Long Fox. The name is one that Bristol is justly proud of, and stands for a rare combination of scientific thought and untiring energy devoted to clinical services and benevolent actions. Owing to the fact that he made a special study of nervous diseases in general, and of those connected with the sympathetic system in particular, it seems probable that, had he been living to-day, Dr. Fox would have been interested in the subject of anæsthesia, seeing how intimately connected some of its phenomena are with the functions of the involuntary nerves.

When a medical student I once had the privilege of being present during a consultation on a patient with symptoms of raised intracranial pressure which Dr. Long Fox diagnosed as due to a cerebral tumour. He expressed the view that trephining might relieve the distress, but that the patient would probably be unable to stand the necessary anæsthetic, which would undoubtedly have been chloroform. Little did I think, on that occasion, that at a future date, forty years thence to be exact, it would fall to my lot to discuss with you, under the ægis of Dr. Fox's memory, the problem of making bad risk operations safer, and the importance, to both surgery and anæsthesia, of the clearest understanding between the specialists concerned.

We live in an age of rapid progress, and may easily forget how far we have travelled or what we have left behind us, but a comparison of the conditions under which operations were performed in pre-anæsthetic days with those obtaining to-day will help us to appreciate the enormous strides which surgery has been able to make since her association with her faithful handmaiden, anæsthesia.

I cannot think that any useful purpose would be served by an attempt on our part to visualize in detail all the horrors inseparable from surgery in those days, or the terrible scenes witnessed in operating theatres so graphically described by many authors,[1] but we can well imagine that before the introduction of anæsthetics operative surgery must, indeed, have been a brutal and nerve-racking occupation ; a most unlikely parent, one would have thought, for an offspring so sympathetic and kindly as the operative surgery of our acquaintance. It is interesting to note from what depths of ill-favour surgery has been raised to its present high position in the world of science. As evidence, we read that Abernethy regarded operations as having no place in surgery as a science ; also that in 1753 Hunter was strongly advised to study medicine rather than surgery, as being a higher branch of the healing art, and he himself is alleged to have regarded the surgeon as a form of armed savage.

The feelings of the time found expression, in 1774, in the hostile attitude of the students of Freiburg towards von Wüthevehr, whom they threatened to mob for daring to advocate the union of surgery with medicine. About this time schools of surgery were established for the benefit of the army, but in Germany it was still considered the duty of surgeons to shave both officers and men, although in England, in 1745, an Act of Parliament was passed to abolish the degrading association of barbers and surgeons.

It seems almost incredible that under such conditions men could be found to inflict, or patients to bear, the agony which an operation must have involved, and we learn that even such a stalwart as Cheselden could seldom sleep on the night preceding an operation. Some idea of the suffering to which a patient had to submit may be gathered from a letter which Simpson received from a colleague describing his own experience when under the knife. He says: "God and man deserted me, and a black whirlwind of emotion and the horror of great darkness overwhelmed me."

In spite of the existing difficulties quite a lot of surgery was performed, nor was it confined to operations of an emergency type. In the seventeenth century, for instance, Abraham Cyprianus, of Amsterdam, besides doing lithotomy no less than 1,400 times, performed abdominal sections and radical cures for hernia; and Larrey, who is said to have conducted 200 amputations in one day after the battle of Moskowa, claimed, among his successes, a case of removal of loose cartilage from a knee-joint, the wound healing by first intention.

I have not quoted the above facts for their historical interest, but in order to illustrate what was the potential eagerness pent up in surgery ready to expand directly the restraining influence of pain and sepsis could be removed.

With the whole world so urgently in need of some method that could assuage pain and thus render surgery available for everybody, it seems surprising that more notice was not taken of the famous letter written in Bristol by Humphry Davy in 1800, in which he said: "As nitrous oxide appears capable of destroying physical pain, it may probably be used with advantage in surgical operations in which no great effusion of blood takes place."[2] That this valuable suggestion should have been allowed to pass unnoticed was due perhaps to the fact that the invention does not seem to have been demonstrated at any surgical centre, and illustrates the truth of our Medico-Chirurgical motto: "Scire est nescire, nisi id me scire alius sciret," or in other words: "One might as well not know a thing as know it and keep it to oneself." On the other hand, however much we may regret the failure to publish more widely this discovery of the properties of nitrous oxide, we must remember that it was not until 1870 that the gas was obtainable in a compressed state in portable cast-iron cylinders, so that its adoption in surgery would have necessitated the centralization of operative work at institutions where the gas could be made on the premises.

The efforts of Colton, begun in 1863, to popularize the gas in dentistry, failed to meet with much success until the year 1867, when he actually demonstrated the method to an English dentist practising in Paris, and from that moment its popularity quickly spread.

In the meantime the use of ether anæsthesia has gone through the same curious phase of remaining for some years known but not appreciated. Its real introduction to the world dates from the occasion in 1846 when Morton administered ether to a patient, on whom Dr. Warren operated, at the Massachusetts General Hospital, but in a letter to Dr. Warren concerning experiments which he had made with ether at the Massachusetts Hospital ten years earlier, in 1836, Morrill Wyman writes: "We each tested the ether by breathing it from a bottle till it produced unconsciousness, the others watching the different effects upon each. We also experimented upon rats in a glass globe until they were entirely motionless, and often wondered that the treatment did them no harm. But we never thought of trying the sensibility under ether even by pricking with a pin. It was a great oversight."

The same thing occurred in reference to chloroform. Early in 1847 Flourens described its power to produce anæsthesia in animals, but its effect was not tried upon the human subject until some months later, when Simpson experimented upon himself during his search for a more pleasant drug than ether; he soon made known his discovery, and within a short time, as the use of the drug spread in many quarters, disasters began to occur under its influence. The mysterious and dangerous phenomena associated with chloroform narcosis have attracted the attention of workers in a variety of research fields, with the ultimate result that the subject of anæsthesia is becoming daily more logical and better understood by both surgeons and administrators.

These three drugs—chloroform, ether and nitrous oxide—together with certain hypnotics and analgesics, form the armamentaria with which the anæsthetist endeavours to co-operate with the surgeon in a combined effort to prevent surgical shock and to avoid post-operative complications as far as possible; in fact, to leave the patient, after operation, in a condition as nearly normal as circumstances will permit. But the surgeon and anæsthetist, in their search for this goal, will have to follow paths set so close together that it will be necessary for them, in places, to proceed, metaphorically, hand in hand, with the possibility of occasionally treading on each other's toes.

Ordinary cases can be adequately dealt with on routine lines; but the bad risk operation is generally preceded by a consultation between all concerned, and it is no unusual thing to find in such cases that the patient stands the ordeal much better than was expected, leading one to speculate whether the good result which so frequently follows such consultations should be attributed to a modified anæsthesia or to a moderated surgical trauma.

We may be excused if we seem over-ready to blame the surgeon for the evil effects of trauma, when with justice we should rather turn to the failure of our anæsthetic to subdue reflexes and counter the shock caused by manipulation; but we may have been deterred, on the one hand, from using an adequately deep anæsthesia owing to our fear of the depressing result of an over-dose, or we may have omitted to use or to suggest the use of an analgesic. This represents a typical example of the overlapping of responsibilities. As a specific illustration of such overlapping I might quote from my notes of a series of thirty-six abdominal operations performed by the same surgeon, in which a combination of light anæsthesia with gentle manipulation resulted in a remarkable freedom from after-effects, except, however, as regards three of the patients, who vomited and showed symptoms of shock, and in these three the abdominal cavity had been "packed off" with towels of a thick, harsh fabric, quite different from the material used for this purpose in the remainder of the series; I feel that this was an instance of faulty partnership, and that either I ought to have deepened the plane of anæsthesia or the surgeon should have employed an analgesic or softer cloths so as to mitigate trauma.

As a matter of fact, the problem of avoiding shock has yielded in a most satisfactory manner to modern devices, but there has not been the same degree of improvement as regards chest complications; and to introduce the topic before a gathering of this nature, with surgeons present, and where discussion is not expected, may be likened to waving a red rag in front of a tethered bull. In reference to anæsthetic risks Sir Benjamin Richardson held the conviction that there was a section of the community, about 1 in every 3,000, which consisted of people ever ready and willing to die, whom he designated as the "Morituri." Although we may hold a more optimistic view nowadays of the attitude of patients to surgery, nevertheless, an analysis of anæsthetic statistics [3] may well leave us inclined to extend this classification so as to include another section consisting of people always ready to develop pneumonia or bronchitis, to be defined as the "Expectoraturi." It will be agreed that this type of individual, when requiring high abdominal surgery, presents us with some of our most difficult problems, demanding the very closest co-operation between surgeon and anæsthetist, in order that the two may realize that they are complementary to one another; in other words, that the more the former assists by limiting the amount of trauma and of heavy stimuli, the less likely will the latter be to cause damage by administering too much anæsthetic.

The other complication most commonly met with after inhalation anæsthesia is sickness, and is generally attributable to the anæsthetic, although in certain cases it may be the result of surgical conditions, such as peritonitis or distended and paralysed intestine. There are, however, a few people who seem to be almost incorrigible in their tendency to vomit, and who might almost claim yet another place in Richardson's classification; but, with modern methods of anæsthesia the incidence of this sequela has dwindled to comparatively small proportions, a result due partly to less anæsthetic poisoning and partly to improved pre-operative preparation, especially as regards the giving of more nourishment, and the avoidance of drastic purging.

The interest taken in the properties and action of the inhalation anæsthetics by workers in various fields of research has resulted in the accumulation of a mass of valuable data upon which to base the choice of method to be used in any particular case, and the selection will depend upon the following factors: safety during administration, capacity as a relaxant, degree of toxicity, reversibility, pleasantness, and ability to prevent shock. Chloroform provides the best form of anæsthesia of any inhalant, keeping the patient perfectly relaxed and immobile, and its abandonment as a routine anæsthetic must have entailed a heavy sacrifice of comfort on the part of operators, but such a course was inevitable in the interest of the patient.

The first reported death under chloroform, which occurred in January, 1848, in Hanna Greener of Newcastle, was soon followed by many others, with the result that an interest in the subject grew up among the profession and spread to other scientists, including physiologists, pharmacologists and chemists, amongst whom Professor Buckmaster held a prominent position.[4] This interest has, fortunately, never been allowed to flag, but has been extended to every problem connected with anæsthesia up to the present time. Between the years 1864 and 1891 no less than four special commissions were convened to discover the causes and means of avoiding danger with chloroform. The outstanding feature of this drug is undoubtedly its tendency to produce a depressant effect upon the circulatory and respiratory systems, and its poisonous action on the heart muscle either when given in concentrated strength for a short period or in a weak form over a prolonged period. This characteristic makes its safety in administration dependent upon the closest attention to detail, and immediate cessation of operation and institution of remedial measures at the first sign of danger. This can only be possible where there is agreement between surgeon and anæsthetist, delay of a few seconds easily leading to death.

The beautiful relaxation so characteristic of this drug kept it in favour for abdominal surgery in spite of the unenviable reputation it was winning for itself in general, but its popularity received a lasting set-back when in 1903[5] Guthrie proved beyond doubt the toxic effects which could be caused by it. The fact soon became recognized that this danger was inseparable from the use of chloroform, and accounted for the persistent nature of the vomiting which was wont to follow a prolonged anæsthesia under its influence. Surgeons were quick to respond by insisting that the drug should not be employed in cases where the integrity of the liver was under suspicion, some even going so far as to exclude it from their clinics altogether. As long ago as 1915 Mr. Moynihan, as he then was, told me that he refused to allow chloroform to be used in any form in his private nursing home.

In 1897, when Mr. Tyrrell introduced his two bottle Junker apparatus for chloroform and ether, he claimed as one of its advantages a reduction in the frequency of vomiting. In the years 1910 to 1912 Mr. Stock and I, in the Infirmary, found that vomiting and acetonuria varied in proportion with the amount of chloroform used. The strength of vapour was controlled by using Mr. Stock's apparatus, when ether vapour was to be added, otherwise by means of the Vernon Harcourt machine, or by a drop method entailing the counting of each drop. The light plane of anæsthesia resulting from this reduction to a minimum of the amount of anæsthetic vapour administered tended to allow the breathing to become too shallow. This symptom was relieved, in a few instances, by the administration of oxygen with 5 per cent. $CO_2$, as had recently been shown by Henderson[6] to be feasible. The result of these investigations went to show that vomiting and acetonuria varied in direct proportion. In a small series of cases in which I had the assistance of Professor Walker Hall in testing for acetone in the blood and urine of the patients in whom the anæsthetic plane had been kept light the incidence was 2 per cent. compared with 10 per cent. in cases where the corneal reflex had been kept in abeyance throughout.

In 1876[7] Claude Bernard stated that all things living were capable of being anæsthetised, and bearing out this suggestion, a good deal of experimental work has been conducted on lower organisms in order to test the relative toxicity of the different anæsthetic drugs, especially of chloroform, ether and nitrous oxide. In 1922 Mr. Hunter and I,[8] working in the botanical and biological departments of this University,[7] were able to prove, to our complete satisfaction, that in every organism we dealt with the toxicity of chloroform was infinitely greater than that of ether, the stimulating phase was shorter and less pronounced, and the paralysing effects were more marked and more slowly recovered from; these results seem to correspond with the phenomena met with clinically.

The paralysing effect produced by chloroform upon the muscles of the abdominal wall naturally meant a degree of relaxation which rendered manipulation easy and free from trauma, and led to the belief that this drug had a special property for preventing shock; but as this involved an undesirable degree of toxicity, it became obvious that the disadvantages outclassed the advantages, with the result that the drug gradually came to be replaced wholly or in part by other agents.

Towards the end of last century Sir Benjamin Richardson, speaking with an intimate knowledge of the action of anæsthetic agents, expressed the opinion that general anæsthesia must eventually give way to local. The tendency to-day is in that direction.[9] The replacement of chloroform by the less toxic ether, and later the attempt to substitute for ether the less poisonous nitrous oxide, may be regarded as parts of the scheme. In this Bristol centre the subject was brought before the profession by Dr. John Freeman at meetings of the Medico-Chirurgical Society in 1895 and 1896. In this neighbourhood, as elsewhere, it was not long before surgeons yielded to conviction.

It may not be unreasonable to claim for ether that it has proved itself a really important factor in the recent advances of surgery. By reason of its safety and flexibility it can, within certain limits of time, be administered in strengths sufficient to subdue or abolish deep reflexes which, if allowed to remain active, would lead to the development of shock and by this means extensive operations may be rendered comparatively harmless. It can be used in practically every operation and in almost every type of patient.

When employed in a closed inhaler ether enables the administrator to exercise with ease complete and instant control over the depth of anæsthesia, but after a time it is apt to increase the movements of respiration and the secretion of mucus to such an extent that abdominal relaxation is no longer possible to obtain. This characteristic led to the adoption of either the open or the perhalation method of administration.

The effects produced by these two methods differ in important essentials, which must be carefully considered before deciding which to employ. The perhalation system, by yielding a deeper and more typically etheric form of anæsthesia, is the easier from the point of view of the administrator, but has the drawback of delivering a vapour chilled below the temperature of the surrounding air, whereas with the frankly open method the reduction of temperature is negligible. In the opinion of many experienced anæsthetists the incidence of lung complications following open ether is at least as great as that associated with closed methods, but these statistics are vitiated by the fact that the term "open ether" is generally used in reference to the perhalation system.

The strictly open method gives a narcosis too light for abdominal surgery in muscular subjects, and may need to be subsidized by the injection of an analgesic, or by the use of pre-operative medication in order to secure relaxation; but it has the advantage of leaving the patient only slightly saturated with the drug, and therefore not prone to prolonged sequelæ. My reason for dilating in some detail upon this part of my subject is, not because I intend to bring forward the usual statistics, proving the superiority of one form of administration over all others, but because my experience in some thousands of cases has taught me, above all things, the inestimable value of the surgeon's co-operation. Where gentle manipulation is not possible, or where a hypnotic or local analgesic is not available, the method may prove unsuitable for abdominal work.

Speaking generally, ether may be said to afford good protection against surgical shock; but this term is a comprehensive one, means any degree of lowered vitality following operation, and should be understood to include all states and degrees of lowered blood-pressure, reduced temperature or impaired resistance to infection. The cause of shock may lie either in surgery or in anæsthesia, and the remedy be a question for consideration of the partnership.

From the anæsthetist's point of view, the course to be taken lies between his Scylla and Charybdis. Too light a narcosis leads to shock by rendering gentleness of manipulation impossible, and too deep an anæsthesia may produce shock through the poisonous effect of the drug itself. In a series of 100 cases at the Bristol Royal Infirmary in 1906, during an investigation into the respective values of the deep and light types of anæsthesia, it was found that the average loss of temperature after operation was invariably greater after deep than after light narcosis, the degree of anæsthesia being determined by the absence or presence of the corneal reflex. On a subsequent occasion, in 1922, Dr. Todd kindly investigated the opsonic index at intervals during ether administration; in each case, after an initial small rise, there was a drop of from 20 to 30 per cent. after ether had been administered for half an hour. This effect upon the phagocytic activity of leucocytes was described by Hamburger[10] in connection with chloroform, and by Graham[11] in connection with ether.

The above clinical experiences have been quoted here because they agree with the findings of certain experimental workers.

Dr. Frank Mann,[12] of the Mayo Clinic, investigating the effect of various ether vapour tensions upon vascular reflexes, found that the best tensions for operative work, in the dog, lay between 38 and 45, and that the corneal reflex was very active with tensions between 36 and 40, but never persisted with tensions over 45. He found that the blood-pressure was always markedly depressed when the ether tension reached 48 or 50. He adds that with the lower tensions, 36 to 45, any depression is quickly recovered from, even after the animal has been kept under ether for some hours ; but that with the higher tensions, 48 to 55, the blood-pressure is slow to recover after the ether is withdrawn and the animal allowed to " come round."

The tendency of ether inhalation to be followed by chest complications makes it advisable that in certain types of operations its use should be limited as far as convenient; it is in this connection that nitrous oxide has proved of such great service. It seems almost incredible to-day that at one time this gas, $N_2O$, was thought to be a poison of the most virulent nature. In *The Considerations on the Production of Fictitious Airs*, published by Dr. Beddoes, 1794–1796, a paper on nitrous oxide appeared, in which the author, S. Latham Mitchill, stated that this gas was so poisonous that a single inhalation of it would destroy life at once, and that it was actually the virus of the fevers and plague. In spite of this warning Davy had the courage to test the truth of the statement by inhaling one deep inspiration of the product, and being unaffected was thus assured that the allegation was false.

The reputation for safety which gas anæsthesia enjoys in short cases does not apply with equal force in the case of prolonged administration, there being a tendency for progressive anoxæmia to develop in spite of the addition of oxygen.

The subject was scientifically investigated by Brown, Lucas and Henderson,[13] who drew the conclusion that "surgical anæsthesia (*i.e.* relaxation) cannot be produced by $N_2O$ and $O_2$ under pressure up to two atmospheres, that surgical anæsthesia with $N_2O$ depends on anoxæmia as well as cerebral depression, that anoxæmia is unavoidable, and that this must limit its use and increase its dangers." Even Davy, during the many months he spent investigating the properties of nitrous oxide, worked out the percentage of oxygen necessary to be mixed with nitrous oxide in order to sustain life, and found that there was a limit to the length of time any animal could be kept alive in this gas. These experiments were conducted with great care, the animals being passed through a medium of water or mercury into the jar containing the gases, so that the percentage of these should not be vitiated by the introduction of air carried in by the animals' fur. Davy assures us that the immersion caused the animals no, or only temporary, inconvenience !

In our own early experiences with nitrous oxide we were puzzled by this anoxæmia, it being easy to keep patients fully anæsthetized for a while, but obviously dangerous to attempt to maintain relaxation beyond ten minutes or so even with a liberal supply of air or oxygen.

In 1906 I used gas and air in a number of cases of midwifery, but found it impossible to produce, with safety, sufficient relaxation during the second stage.

Patients, however, were allowed to help themselves to the gas during pains, and trouble never arose from this arrangement.

It is important that the partnership should agree on this point, otherwise the surgeon may expect deep anæsthesia to be kept up without the assistance of premedication, or local analgesics, or ether. Even with ethylene and oxygen in equal proportions some ether is required for complete relaxation.

Before embarking on a gas anæsthesia the administrator should understand how much he is to look to ether or to a preliminary hypnotic and how much to a nerve blocking for the necessary assistance.

Crile, in expounding his famous anoci-association method, suggested that the surgeon who finds relaxation unsatisfactory should look to his local analgesia rather than to the anæsthetist for rectification of the fault. I would suggest that the surgeon should not hesitate to tell the anæsthetist what is needed, remembering that nothing hurts the feelings of the anæsthetist more than a profane silence on the part of the surgeon; it signifies a state of tension in the partnership.

As regards the frequent occurrence of lung complications after high abdominal operations, although we must attribute a large portion of the blame to the anæsthetic, we cannot afford to overlook the element of paralysis affecting the nerves which govern the movements of the lower intercostals and diaphragm, and at the same time innervate the peritoneum covering the gall-bladder, the diaphragm and the pyloric end of the stomach, and one is led to wonder whether there will ever be discovered a surface

Q

anæsthetic with so low a toxicity that its application to a large surface such as that of the peritoneum will be safe. Percaine may bring us a step farther in this direction. Such a drug would revolutionize anæsthesia in many respects.

In connection with local anæsthetic drugs the work done in this University some years ago by Professor Francis and the experiments which Mr. Eric Watson-Williams[14] conducted here in 1925 represent the sort of investigations from which we may hope, some day, to derive such a boon to surgery.

The subject of basal anæsthetics is much to the fore at present, and the possibilities of developing the chemistry of the barbiturates along rational lines has been explained in this Department as recently as March, 1931, by Dr. Nierenstein, with demonstrations by Professor Brocklehurst,[15] with an account of their pharmacological aspects by Mr. A. L. Taylor. The wholesome result of such scientific help to the profession is that it enables us to realize how desirable it is that we should try to include in our partnership workers in academical departments.

In England to-day we occupy a particularly fortunate position as regards anæsthesia, with ether and gas holding the central position and with hypnotics and local analgesia, including spinal, well established, so to speak, on either side. It is true that manufacturers report that there still exists a large demand for chloroform, but as far as the large teaching clinics are concerned it is used chiefly as an auxiliary, and chloroform anæsthesia proper has become very rare.

Local methods are coming into their own, but in a country like this, where there are so many expert inhalation anæsthetists, such methods can be kept for occasions where they offer some real advantage to the patient, and where less tedious methods are contra-indicated for some reason.

The decision as to which type of anæsthesia to employ is at times extremely difficult, especially as in desperate cases the problem of risk is apt to be viewed from opposite angles by the surgeon and the anæsthetist. The question is often asked: "Can the patient stand an anæsthetic?" But seeing that there is practically no condition in which some form of anæsthetic cannot be taken, it might be more logical to inquire whether the patient could survive an anæsthetic plus the proposed operation. In other words, we have to remember that anæsthesia does not offer a complete barrier to the shock of surgery.

The same difficulty arises when spinal analgesia is advocated in such cases. There is a tendency to forget that a patient may be too ill for spinal and yet able to take a general anæsthetic with comparative safety.

May I tender my thanks to you, Mr. Vice-Chancellor, for so kindly presiding over this meeting, and to my audience for so patiently listening to me and for so politely remaining in the stage of consciousness; and explain that in order to attune my remarks, as far as possible, to the spirit which I believe should be displayed in these lectures, I have omitted the optimistic accounts of new drugs and infallible methods coming to us almost daily from outside, and have attempted to produce a few impressions based upon work done in home circles.

I feel that Dr. Long Fox would have preferred us to relate what we ourselves have done, rather than what we may have heard or read from outside sources.

REFERENCES.

[1] *Vide* address by Warren, *Trans. Amer. Surg. Assoc.*, 1897, xv. 1 *seq.*

[2] *Davy, Works of*, London, 1839, iii. 196.

[3] Featherstone, "Inquiry into Causation of Post-operative Pneumonia," *Brit. Jour. Surg.*, 1924–5, xii. 487.

[4] *Proc. Roy. Soc. B.*, 1917, lxxix. 555.

[5] *Lancet*, 1903, ii. 10.

[6] Yandell Henderson, *Amer. Jour. Physiol.*, 1908, xxi. 126.

[7] Bernard, *Leçons sur les phénomènes de la vie communs aux animaux et végétaux*, Paris, 1878.

[8] Flemming, *Brit. Med. Jour.*, 1922, ii. 22.

[9] Flemming, *Proc. Roy. Soc. Med.*, 1910–11, iv., Sect. Anæsth., 1.

[10] Hamburger, *Archives Néerlandaises: des sciences exactes et naturelles*, Series 3B, 1911, 1.

[11] Graham, *Jour. Amer. Med. Assoc.*, 1910. liv. 1,043.

[12] Mann, *America: Year-book of Anæsthesia and Analgesia*, 1917, p. 98.

[13] Brown, Lucas and Henderson, *Jour. Pharm. and Exper. Therapeutics*, xxxi. 269.

[14] Williams, *Bristol Med.-Chir. Jour.*, 1925, xlii. 164.

[15] *Proc. Roy. Soc. Med.*, 1930–31, xxiv., Sect. Anæsth., 861.

